# Personalization of medical device interfaces: decreasing implant-related complications by modular coatings and immunoprofiling

**DOI:** 10.2144/fsoa-2020-0074

**Published:** 2020-07-30

**Authors:** Nihal Engin Vrana, Kaia Palm, Philippe Lavalle

**Affiliations:** 1 Spartha Medical, 14B Rue de la Canardiere, 67100, Strasbourg, France; 2 INSERM UMR1121 “Biomaterials & Bioengineering” 11 Rue Humann, 67000, Strasbourg, France; 3 Protobios Llc, Mäealuse 4, 12618 Tallinn, Estonia; 4 Universite de Strasbourg, Faculté de Chirurgie Dentaire, 1 Porte de l'Hospital, 67000, France

**Keywords:** adverse immune reactions, antimicrobial coatings, immunoprofiling, implants, nosocomial infections

## Personalized medicine & medical devices

The organ/tissue function replacement using implantable medical devices has become a pillar of modern medicine with a considerable level of success. Cochlear, orthopedic and dental implants are some of the examples of implantable medical devices with more than several million implantations per year. However, such an increased volume of use has also brought about an increased level of complications. One such complication is infections around medical devices, particularly in hospital settings where a significant portion of infections called ‘nosocomial infections’ is closely related with implanted devices. Although there are certain indications such as smoking, presence of chronic systemic diseases and so on, there is no current method available to predict such medical complications.

One potential way to handle this problem follows the recent developments in personalized medicine. Currently in the field of oncology and in pharmaceutics in general, patient-specific precision medicine [1] techniques are taking hold. The most basic approaches are based on demographic correlations (such as lifestyle-related stratification of the therapies for improved efficacy), but more advanced techniques look at the gene expression level (such as determination of the expression of HER-2, a specific growth factor receptor which is the target of some breast cancer drugs). In the field of implantable devices, the personalization up to now has taken the form of application of additive manufacturing techniques for obtaining personalized implants with better anatomical fits for a given patient. Beyond this, with such techniques it is also possible to print personalized surgical instrumentations and personalized models for pre-operation decision making [2]. Accompanying such efforts, advances in imaging technologies have resulted in dedicated systems for personalized planning of implant positioning (such as tools that enable virtual implant positioning on 3D constructed images of the defect/damaged area). However, there is another important determinant of the success of implants, which is the immune response to the implanted material, as an implant is generally considered by the body as a foreign object [3]. The extent, intensity and the severity of such immune reactions are highly personalized also. Thus, it is equally important not only to create personalized interfaces between the implants and the host tissues in a patient specific, but also organ or tissue-specific manner.

## Ways of achieving personalization

Interface personalization can be achieved in several ways such as patient-specific modification of surface properties of implants including wettability, roughness, nano/microscale topography [4], chemistry and so on [5]. Another way to achieve interface personalization is the incorporation of delivery systems for cytokines that will regulate the implant microenvironment as a function of the expected immune response of the patient. This can be done by a single cytokine that is highly relevant for a given tissue [6] or via cytokine cocktails [7]. A third option is to create patient-specific coatings on implant materials that will act as an optimized interface. Such coatings should be multifunctional [8] in the sense that they should not only provide an optimized microenvironment but they should also incorporate preventive measures such as antimicrobial properties [9]. This will mean surfaces that will provide amenable properties for the tissue-specific cell type to adhere and cover the implant surface to facilitate integration, and that can resolve the initial inflammation in a pro-regenerative manner to diminish adverse immune reactions and the associated collateral damage. Moreover, these surfaces should have the necessary components to prevent opportunistic bacterial, fungal and maybe even local viral infections.

Immunoprofiling can be achieved by the quantification of the soluble factors in the patient’s blood, or by *in vitro* evaluation of the behavior of the isolated patient immune cells. However, in order to delve into the background of an individual’s adaptive immune response to a given material, antibody-level information is necessary. For this end, use of mimotope variance analysis [10] provides a unique opportunity to not only determine the generic responses to new biomaterials, but also patient-specific response to biomaterials, which can guide the development of immune-compatible, patient-specific implant surfaces. Such immunoprofiling based pre-implantation and postimplantation diagnostics can improve the functional outcomes of different implants by aiding in the implant type selection and can also significantly decrease the implant-related complications.

The coupling of immunoprofiling with immunomodulatory surface modifications would provide us with an arsenal to resolve tissue replacement-related problems in a patient-specific manner without searching for new materials for each new situation. This will also broaden the capacities of the multiscale coatings from the resolution of the known complications to have immunomodulation in their vicinity and also protective solutions in conditions such as the ongoing COVID-19 outbreak.

## References

[1] Petkova E, Park H, Ciarleglio A, Ogden RT, Tarpey T Optimising treatment decision rules through generated effect modifiers: a precision medicine tutorial. BJPsych. Open 6(1), e2 (2020). 10.1192/bjo.2019.85 PMC7001471 31791433

[2] Hart A, Hart A, Panagiotopoulou V, Henckel J Personalised orthopaedics–using 3D printing for tailor-made technical teaching, pre-operative planning and precise placement of implants. Orthop. Prod. News 178, 22–26 (2017).

[3] Vrana NE Immunomodulatory biomaterials and regenerative immunology. Future Science 2(4), FSO146 (2016). 10.4155/fsoa-2016-0060 PMC5242206 28116128

[4] Dollinger C Controlling incoming macrophages to implants: responsiveness of macrophages to gelatin micropatterns under M1/M2 phenotype defining biochemical stimulations. Adv. Biosyst. 1, 1700041 (2017).

[5] Mas-Moruno C, Su B, Dalby MJ Multifunctional coatings and nanotopographies: toward cell instructive and antibacterial implants. Adv. Healthc. Mater. 8, 1801103 (2019). 10.1002/adhm.201801103 30468010

[6] Clauder F Multifunctional coatings combining bioactive peptides and affinity-based cytokine delivery for enhanced integration of degradable vascular grafts. Biomater. Sci. 8, 1734–1747 (2020). 31998886 10.1039/c9bm01801h

[7] Ščigalková I The effect of healing phenotype-inducing cytokine formulations within soft hydrogels on encapsulated monocytes and incoming immune cells. RSC Adv. 9, 21396–21404 (2019). 10.1039/c9ra02878a PMC9066154 35521319

[8] Raphel J, Holodniy M, Goodman SB, Heilshorn SC Multifunctional coatings to simultaneously promote osseointegration and prevent infection of orthopaedic implants. Biomaterials 84, 301–314 (2016). 26851394 10.1016/j.biomaterials.2016.01.016 PMC4883578

[9] Knopf-Marques H Multifunctional polymeric implant coatings based on gelatin, hyaluronic acid derivative and chain length-controlled poly(arginine). Mater. Sci. Eng. :C 104, 109898, (2019). 10.1016/j.msec.2019.109898 31499960

[10] Sadam H Prostaglandin D2 receptor DP1 antibodies predict vaccine-induced and spontaneous narcolepsy type 1: large-scale study of antibody profiling. EBioMedicine 29, 47–59 (2018). 29449194 10.1016/j.ebiom.2018.01.043 PMC5925455

